# High frequency optogenetic activation of inputs to the lateral amygdala forms distant association with foot-shock

**DOI:** 10.1186/s13041-020-00587-4

**Published:** 2020-03-20

**Authors:** Fei Li, Chun-Hui Jia, Jun Huang, Guo-Qiang Bi, Pak-Ming Lau

**Affiliations:** 1 CAS Key Laboratory of Brain Function and Disease, and School of Life Sciences, University of Science and Technology of China, Hefei, 230026 Anhui China; 2 CAS Center for Excellence in Brain Science and Intelligence Technology, University of Science and Technology of China, Hefei, 230026 Anhui China

**Keywords:** Lateral amygdala, Optogenetic stimulation, Distant fear conditioning

## Abstract

**Aim:**

A hallmark of classical conditioning is that conditioned stimulus (CS) must be tightly coupled with unconditioned stimulus (US), often requiring temporal overlap between the two, or a short gap of several seconds. In this study, we investigate the temporal requirements for fear conditioning association between a strong artificial CS, high-frequency optogenetic activation of inputs into the lateral amygdala of rats, and a foot-shock to the animal with delays up to many minutes.

**Methods:**

AAV-oChIEF-tdTomato viruses were injected into the auditory cortex and the medial geniculate nucleus of rats. An optical fiber was implanted just above the lateral amygdala of the animal. Optogenetic high-frequency stimuli (oHFS; containing five 1-s trains of 100 Hz laser pulses) were delivered to the lateral amygdala, before or after (with varying intervals) a foot-shock that elicits fear responses in the animal. Pre-trained lever-press behavior was used to assess the degree of fear recall by optogenetic test stimuli (OTS; 10 Hz for 2 min) 24 h after the association experiment.

**Results:**

In contrast to the tight temporal requirement for classical conditioning with paired optogenetic moderate-frequency stimuli (oMFS; 10 Hz for 20 s) and foot-shock, oHFS followed by foot-shock with a 5-min or even 1-h (but not 3-h) interval could successfully establish an association to be recalled by OTS the next day. Meanwhile, foot-shock followed by oHFS with a 5-min (but not 1-h) interval could also establish the conditioning. Thus, distant association may be formed between temporally distant stimuli when the CS is strong.

Animals live in a complex environment and experience many events of different significance every day. Learning the causal or temporal relationship, i.e., forming association, between important events is crucial for the animal to adapt to the environment and survive. In classical conditioning, long-term associative memory is formed after repeated pairing of the conditioned stimulus (CS) and the unconditioned stimulus (US) [1, 2], often with the US presented before the end the CS presentation as in the case of delay conditioning, or with the US following the CS by short gaps of several seconds as in the case of trace conditioning [3, 4]. When the gap is too long, e.g. beyond 30 s, the mild CS and the strong US are considered “unpaired”, and association cannot be formed between them [4]. Intuitively, the significance of sequential events could also have an impact on the formation of association. However, it has remained unclear whether a stronger (and perhaps more significant) “CS”-like stimulus can form “distant” association with the US separated by longer gaps.

The lateral amygdala (LA) has been established as a key brain area for auditory fear conditioning [5–7]. In a recent study [8], Nabavi et al. used optogenetic stimulation to axonal inputs into the LA from the auditory cortex (AC) and the medial geniculate nucleus (MGN), to investigate its association with the US (foot-shock) and how this association could be erased by long-term depression (LTD) and reestablished by long-term potentiation (LTP). Inspired by this paradigm, we expressed a light-activated channelrhodopsin ChIEF via adeno associated virus (AAV) in the AC and the MGN of Sprague-Dawley rats, and implanted an optic fiber above the LA for optogenetic stimulation of axonal inputs in this area from the virally infected neurons (Fig. 1a). Optogenetic stimulation and foot-shocks were used for association training, followed by fear recall testing with a pre-trained lever-press task 24 h later [9, 10] (Fig. 1b, Additional file 1: Materials and Methods).

**Fig. 1:**
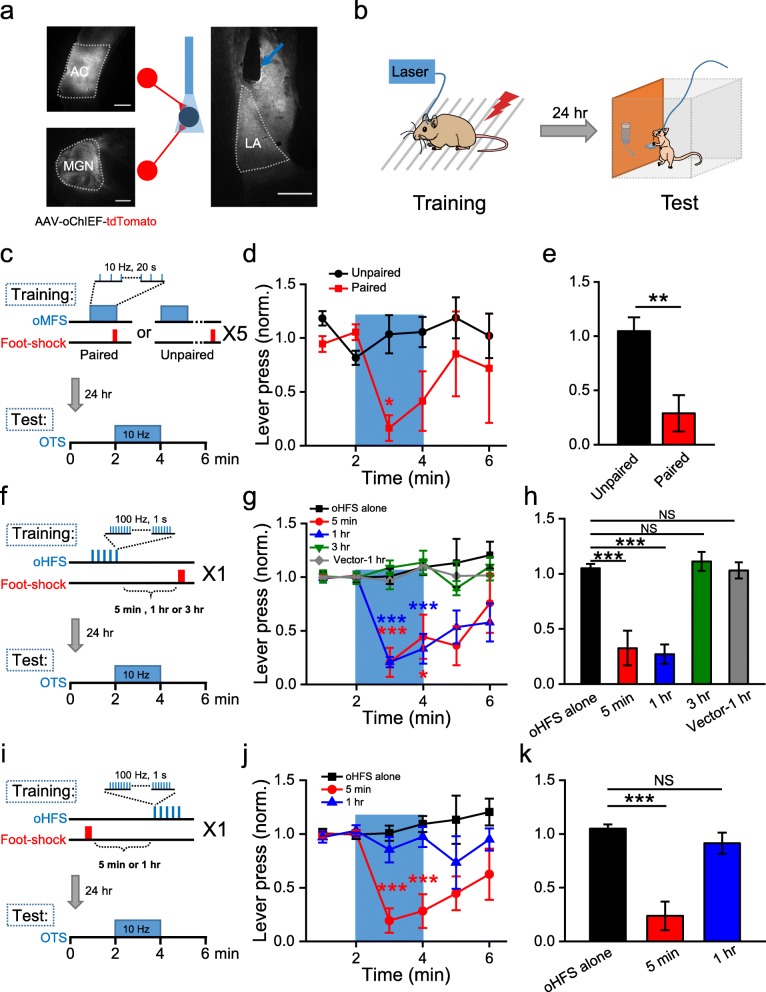
Association between optogenetic stimulation and foot-shock. **a** Expression of oChIEF-tdTomato in the AC and the MGN (left), as well as the LA (right) 4 weeks after viral injection. Blue arrow indicates position of implanted optic fiber. Scale bars: 500 μm. **b** Behavioral paradigms of associative fear training (left) and recall test (right). **c**. Fear training using oMFS paired or unpaired with foot-shocks (repeated every 3 min for 5 times). Fear response was assessed in the lever-pressing box 24 h later with OTS. **d** Normalized number of lever presses (shown in 1-min bin) from lever-pressing tests 24 h after fear training. Lever pressing was significantly inhibited by OTS (blue area) in the paired group (*n* = 4) but not in the unpaired group (*n* = 4). * indicates *P* < 0.05; one-way repeated ANOVA followed by Tukey’s multiple comparison test. All error bars in this and subsequent figures are SEM. **e** Mean normalized number of lever presses during the 2 min of the OTS. Significant reduction in lever pressing was found in paired group (*n* = 4) compared with the unpaired group (*n* = 4). ** indicates *P* < 0.01; Student’s t-test. **f**-**h** Similar to **c-e**, but for fear training with oHFS followed by a 3-s foot-shock with a delay of 5 min, 1 h or 3 h. Significant reduction in lever pressing was found in the 5-min group (*n* = 6) and 1-h group (*n* = 7), but not in the oHFS alone group (*n* = 6) or 3-h group (*n* = 6) or vector-1-h control group (*n* = 6). * indicates *P* < 0.05; *** indicates *P* < 0.001; one-way repeated ANOVA (**g**) and one-way ANOVA (**h**) followed by Tukey’s multiple comparison test. **i**-**k** Similar to **f**-**h**, but for fear training with oHFS 5 min and 1 h after the foot-shock. Significant reduction in lever pressing was found in the 5-min group (*n* = 7) but not in the 1-h group (*n* = 6). The same data for the oHFS alone group in **g**-**h** is shown here again as a control. *** indicates *P* < 0.001; one-way repeated ANOVA (**j**) and one-way ANOVA (**k**) followed by Tukey’s multiple comparison test

With this system, we first confirmed that optogenetic moderate-frequency stimuli (oMFS; 10 Hz for 20 s) paired with foot-shocks delivered at the end of each stimulation train (repeated every 3 min for 5 times) resulted in associative fear memory, which when recalled by a 2-min train of 10 Hz optogenetic test stimuli (OTS) the next day, interrupted lever-press behavior (Fig. 1c-e). In contrast, “unpaired” oMFS with foot-shocks delivered at least 1-min afterwards could not establish such association: OTS the next day did not affect lever-press behavior (Fig. 1c-e). These results are consistent with previous findings [8], and suggest that optogenetic stimulation to the LA input projections could be used as an artificial “CS” for classical fear conditioning.

To evaluate the effects of a stronger artificial “CS” for potential “distant association”, we chose optogenetic high-frequency stimuli (oHFS; 100 Hz light pulses for 1 s, repeated 5 times every 3 min), followed by a single 3-s foot-shock with a delay of 5 min, 1 h or 3 h after the oHFS (Fig. 1f). In the lever-pressing test the next day, the same 10 Hz OTS was used because it is closer to the physiological firing frequency of LA neurons during natural fear recall [11]. Intriguingly, animals trained at 5-min and 1-h (but not 3-h) oHFS-shock intervals exhibited apparent fear recall behavior, with lever press significantly inhibited by OTS during the test (Fig. 1g-h). Therefore, a stronger CS-like stimulus could indeed establish distant association with the US delivered even after 1 h later.

As a control experiment, we used rats with AAV-hSyn-tdTomato injected in the AC and the MGN. These animals were trained with oHFS followed by foot-shock 1 h later (Fig. 1g-h, vector-1-h group). No fear response was detected during fear test the next day in these animals, in contrast to the significant reduction of lever pressing in the oChIEF expressing 1-h group (Fig. 1g-h). Thus, the behavioral responses are most likely due to optogenetically evoked neuronal activity in the LA. In another control experiment, we trained the animal using a similar paradigm but replaced the oHFS with oMFS (10 Hz for 50 s, total 500 pulses to match the pulse number in oHFS). We found that even when the foot-shock followed the prolonged oMFS by only 10 s, no fear response was recalled by OTS the next day (Additional file 1: Fig. S1). Thus, the high stimulation frequency in oHFS was crucial for such distant association.

We further tested “backward” distant association by delivering the foot-shock first, followed by the oHFS at different intervals (Fig. 1i). Intriguingly, fear response was observed in the lever-pressing test the next day for animals with 5-min shock-oHFS interval but not those with 1-h interval (Fig. 1j-k). Thus, the distant association appeared to be bi-directional and temporally asymmetric, reminiscent of bi-directional trace conditioning observed in previous studies, although the latter has a much shorter timescale [12].

How could the oHFS form distant association with the foot-shock? It has been well established that synaptic plasticity such as NMDA receptor-dependent LTP in the LA plays an important role in fear conditioning [8, 13, 14]. With systemic NMDA receptor blocker MK801 injection before association training, we found that distant association at 1-h oHFS-shock interval could not be formed (Additional file 1: Fig. S2), indicating that NMDA receptor and activity-dependent synaptic plasticity are likely involved in this process. However, neuronal activation and NMDA receptor signaling in the current paradigm occur on the timescale of milliseconds to seconds, much shorter than that for distant association. One possible scenario is that the oHFS caused long-lasting changes in neuronal excitability and network activation, which act as a long “trace” to couple with the distant US. Along this line, comparing how different patterns of optogenetic stimuli elicit different levels of acute and chronic neural activation in the LA in vivo and in vitro could provide important insights. Another possibility is that the oHFS (perhaps via strong activation of NMDA receptors) initiated synaptic plasticity signals that lasted for minutes to hours and “consolidated” by subsequent US, through a mechanism analogous to “synaptic tagging” [15]. Whereas these possibilities remain to be investigated in future studies, the existence of distant association beyond the usual timescale of classical conditioning demonstrates the complexity of associative learning, and suggests new mechanisms underlying learning and memory as well as related brain disorders.

## Supplementary information

**Additional file 1: Materials and Methods**, **Fig. S1** Distant association could not be formed with oMFS followed by foot-shock, **Fig. S2** Systemic administration of MK801 before fear training blocked distant association.

## Data Availability

All data presented are available upon request.

## Abbreviations

CS: Conditioned stimulus
US: Unconditioned stimulus
AC: Auditory cortex
MGN: Medial geniculate nucleus
LA: Lateral amygdala
oMFS: Optogenetic moderate-frequency stimuli
oHFS: Optogenetic high-frequency stimuli
OTS: Optogenetic test stimuli
NMDA: N-methyl-D-aspartate
LTP: Long-term potentiation
LTD: Long-term depression
