# Supplementary material for: High frequency optogenetic activation of inputs to the lateral amygdala forms distant association with foot-shock
Source: Mol Brain. 2020 Mar 20;13:44. doi: 10.1186/s13041-020-00587-4 (PMC7082919; doi:10.1186/s13041-020-00587-4)
Supplement: Supplementary file 1 — Additional file 1: Materials and Methods, Fig. S1 Distant association could not be formed with oMFS followed by foot-shock, Fig. S2 Systemic administration of MK801 before fear training blocked distant association. [file 13041_2020_587_MOESM1_ESM.docx]

Additional file 1

**High frequency optogenetic activation of inputs to the lateral amygdala forms distant association with foot-shock**

Fei Li^1^, Chun-Hui Jia^1#^, Jun Huang^1#^, Guo-Qiang Bi^1,2^* and Pak-Ming Lau^1^*

*Correspondence: gqbi@ustc.edu.cn or [plau@ustc.edu.cn](mailto:plau@ustc.edu.cn)

**This file includes:**

Materials and Methods

Fig. S1

Fig. S2

**Materials and Methods**

**Animals:**

All animal experiments were conducted following protocols approved by the Animal Experiment Committee at the University of Science and Technology of China. Male Sprague-Dawley rats were housed under a 12-hour light/dark cycle, with *ad libitum* access to food and water. The rats were about 8 weeks old when used for experiments.

**Virus injection and surgery:**

Rats (8 weeks old) were anesthetized with 2% pentobarbital sodium (60 mg/kg, Merck) and were mounted in a stereotaxic frame. The body temperature was kept around 37.5 °C using a heating pad during surgery and recovery from anesthesia. AAV viruses (Shanghai Taitool Bioscience) carrying hSyn-oChIEF-tdTomato or hSyn-tdTomato (vector-only, for control experiments) constructs were injected into the medial geniculate nucleus (AP: -5.1 mm and -5.7 mm; ML: 2.9 mm; DV: -5.5 to -6.5 mm) and the auditory cortex (AP: -5.7 mm; ML: 4.8 mm with a 20-degree angle; DV: -4.5 to -5.7 mm). A total of 400 nl of virus was injected for each site at a speed of 40 nl / min. A multimode optical fiber of 200 μm dimeter (NA = 0.37, Suzhou CooCore Photoelectronic Technology) was implanted above the lateral amygdala (AP: -3.3 mm; ML: 4.2 mm; DV: -7 mm with a 7-degree angle) and was secured on the skull with four screws and dental cement. Animals were used for behavioral experiments 4 weeks after viral injections, when plenty of oChIEF-tdTomato expressions were observed under a fluorescence microscope.

**Behavioral assays:**

**Lever press training.** Animals were trained for 1 week starting from 3 weeks after the surgery. The custom-made lever-pressing box (35 cm Х 35 cm Х 30 cm) was controlled by an Arduino-based custom-program. Water restricted rats (with 2 hours of free water intake daily) were allowed 5 minutes of free exploration in the behavior box before having access to the lever, and every lever press was rewarded with a drop of sucrose solution (40 μl of 10% sucrose, Sinopharm Chemical Reagent Co). The rats were given 40 minutes of lever press training every day.

**Fear training.** Fear training was performed about four weeks after viral injection. Rats had free access to water 24 hours before conditioning training. For optogenetic stimulation, a 473 nm laser (Shanghai Fiblaser Technology) was connected to the pre-implanted optical fiber through another 200 μm diameter multimode optical fiber to deliver light pulses to the LA. For optogenetic moderate-frequency stimuli (oMFS) paired with foot-shock, a 20-sec train of 10 Hz pulses (10-15 mW at the output of the implanted optical fiber, 2-ms pulse width) was co-terminated with the foot-shock (0.8 mA, 2 seconds) and repeated every 3 minutes for 5 times. For the unpaired group, there was an interval (60-120 s, randomly assigned) between oMFS and foot-shock. For distant association, optogenetic high-frequency stimuli (oHFS; five 1-sec trains of 100 Hz light pulses separated by a 3-min interval, 10-15 mW, 2-ms pulse width) were delivered before or after the foot-shock (0.8 mA, 3 seconds). After training, rats were allowed to rest in the box for 5 minutes before being returned to the home cage.

**Fear recall test.** Rats were water restricted for 24 hours before being placed in the lever-pressing box. After two minutes of stable lever press (at least 6 lever presses per minute), optogenetic test stimuli (OTS; 10 Hz for 2 minutes) were delivered through the implanted optical fiber. After light stimulation, another 2-min of post-stimulation lever press behavior was also monitored. The number of lever presses during the 2-min OTS period was counted as a measure of fear response. Rats were allowed to stay in the behavior box for 5 minutes after the fear recall test.

**Histology and imaging:**

After the fear recall test, rats were sacrificed and transcardially perfused with saline followed by 4% paraformaldehyde (Sigma) in PBS. The brains were kept in 4% paraformaldehyde at 4 °C for 24 hours and then 30% sucrose for 72 hours before being sliced into 40-μm coronal sections and imaged under a fluorescence microscope (Olympus MVX10).

**Statistics:**

All values are expressed as mean ± SEM. One-way repeated ANOVA followed by Tukey’s multiple comparison test was used for comparing lever press before and after optogenetic stimulation. Student’s t-test or one-way ANOVA followed by Tukey’s multiple comparison test were used for comparing lever press in experimental and control groups.


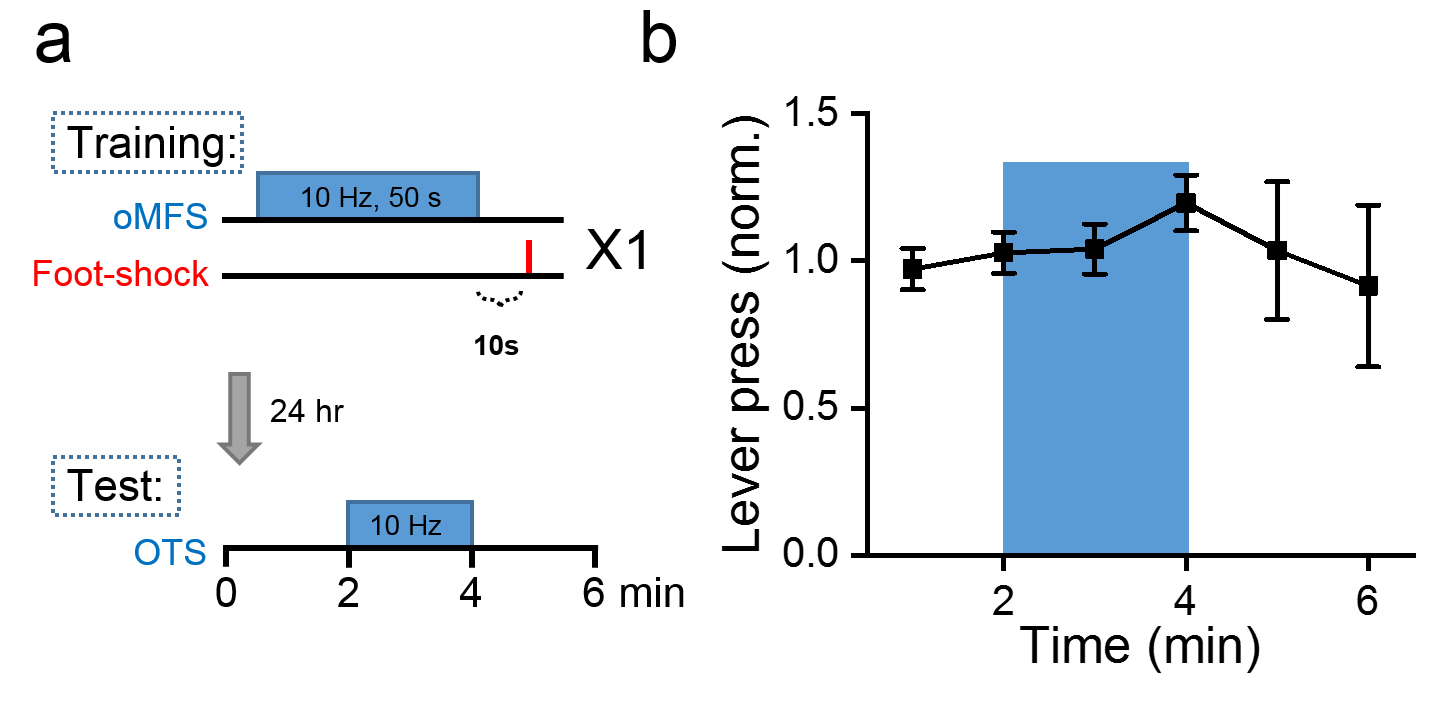


**Fig. S1** **Distant association could not be formed with oMFS followed by foot-shock.**

**a.** Fear training paradigm with prolonged oMFS (10 Hz for 50 seconds) delivered to the LA 10 seconds before a 3-sec foot-shock.

**b.** Normalized number of lever presses 24 hours after oMFS fear training. OTS caused no significant change in lever-pressing (P = 0.91 and P = 0.20 at 3 minute and 4 minute point， respectively, n = 4; one-way repeated ANOVA followed by Tukey’s multiple comparison test).


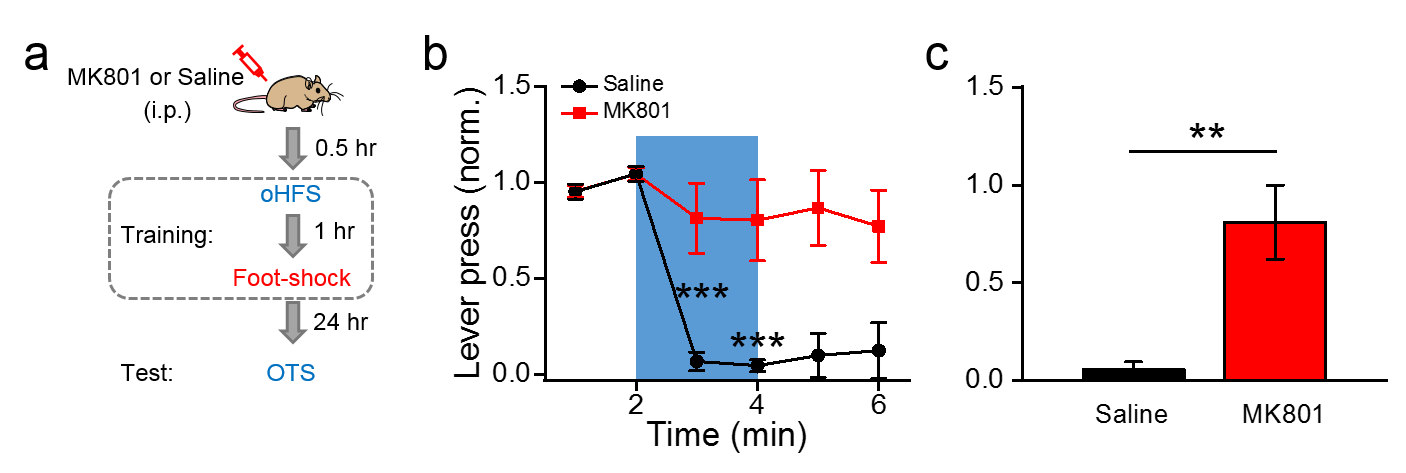


**Fig. S2** **Systemic administration of MK801 before fear training blocked distant association.**

**a.** Experimental paradigm. Rats were intraperitoneally injected with MK801 (0.2 mg/kg, Sigma) or saline 0.5 hour before oHFS, which was followed by foot-shock 1 hour later.

**b.** Results of fear recall tests. Significant reduction in the normalized number lever presses was found in the saline group (n = 4) but not in the MK801 group (n = 6). *** indicates P < 0.001; one-way repeated ANOVA followed by Tukey’s multiple comparison test.

**c.** Mean normalized number of lever presses during the 2 minutes of the OTS. Significant reduction in lever pressing was found in the saline group (n = 4) compared with the MK801 group (n = 6). ** indicates P < 0.01; Student’s t-test.
